# Supplementary material for: Asynchronous magnetic resonance elastography: Shear wave speed reconstruction using noise correlation of incoherent waves
Source: Magn Reson Med. 2022 Oct 27;89(3):990–1001. doi: 10.1002/mrm.29502 (PMC9792433; doi:10.1002/mrm.29502)
Supplement: Supplementary file 1 — DATA S1 MATLAB code used to generate the numerical simulations of Figure 1 [file MRM-89-990-s002.zip › k-Wave/helpfiles/example_pr_2D_tr_time_variant_filtering.html]

Attenuation Compensation Using Time Variant Filtering Example (k-Wave)


# Attenuation Compensation Using Time Variant Filtering Example

This example demonstrates how the acoustic attenuation present in the photoacoustic forward problem can be compensated for using time-variant filtering. It builds on the 2D Time Reversal Reconstruction For A Circular Sensor and Attenuation Compensation Using Time Reversal examples.

- Open the file in the MATLAB Editor
- Run the file in MATLAB

For a more detailed discussion of this example and the underlying techniques, see B. E. Treeby "Acoustic attenuation compensation in photoacoustic tomography using time-variant filtering," *J. Biomed. Opt.*, vol. 18, no. 3, p.036008, 2013.

## Contents

- Running the forward simulation
- Attenuation compensation with a fixed cut-off frequency
- Using a time-variant window
- Correcting the signals individually

## Running the forward simulation

The recorded sensor data for an attenuating medium is simulated using `kspaceFirstOrder2D` with the initial pressure distribution set to the Shepp Logan phantom, in the same way as the previous example. A circular Cartesian sensor mask with 200 sensor points is used to record the photoacoustic signals. After the simulation, random Gaussian noise is added using `addNoise` to give a signal-to-noise ratio of 40 dB. The sensor data is then used to reconstruct the photoacoustic image using time reversal. The initial pressure distribution and reconstructed image are shown below. The reconstructed image is very blurred due to the loss of high frequency information, and the magnitude of small features in the image is significantly reduced. This is also visible in the profiles, where the black line is a profile through the initial pressure distribution at x = 0, and the red line is the same profile through the reconstructed image.

  


## Attenuation compensation with a fixed cut-off frequency

In the Attenuation Compensation Using Time Reversal Example, the acoustic attenuation in the forward problem is corrected as part of the time-reversal image reconstruction procedure by reversing the sign of the absorption term in the numerical model, thereby causing the waves to grow rather than decay. For a homogeneous distribution of absorption parameters, it is also possible to compensate for acoustic attenuation directly in the recorded time series (i.e., before image reconstruction) using time-variant filtering. The general idea is that the signals are modified using a filter that depends on both time and frequency. Here this is achieved using a form of nonstationary convolution using the function `attenComp`.

Analogous to attenuation compensation using time-reversal, if the time-variant filter is applied to experimental or noisy data, the high frequency content (where the signal-to-noise ratio is typically quite low) can become amplified and mask the desired features within the signals. To avoid this, the time-variant filter is regularised using a window with a time-variant cutoff frequency. The cutoff frequency can be calculated automatically (as discussed in the following section), or specified manually. In the example below, the cutoff frequency for the window is defined manually at 3 MHz. This matches the cutoff used in the Attenuation Compensation Using Time Reversal Example. The attenuation compensation is then applied to each time series in the sensor data, where the time-variant filter is constructed based on the specified sound speed and attenuation parameters. The attenuation is assumed to follow a frequency power law of the form `alpha_coeff*f^alpha_power`, where `alpha_coeff` is defined in units of [dB/(MHz^y cm)]. When using a fixed cutoff frequency, the compensation is extremely fast to apply.

```
% correct for acoustic attenuation using time variant filtering regularised
% by a Tukey window with a fixed cutoff frequency
sensor_data_comp = attenComp(sensor_data_lossy, kgrid.dt, medium.sound_speed, ...
    medium.alpha_coeff, medium.alpha_power, 'FilterCutoff', [3e6, 3e6]);
```

The sensor data after attenuation compensation is then used to reconstruct the photoacoustic image using time reversal. As shown below, the compensation makes the reconstructed image much sharper, and the magnitude of the edges of the phantom are now much closer to the initial pressure distribution. Choosing a fixed cutoff frequency for the regularising window in this way gives the same correction as using time reversal with attenuation compensation (discussed in the previous example). However, the drawback of choosing the cutoff frequency in this way is that the frequency content of the recorded photoacoustic signals is not normally uniform over time. This results in a compromise between the level of attenuation compensation, and the level of noise in the reconstructed image. In this example, it is clear that noise has been introduced (visible as a rippled texture) that wasn't present in the initial pressure distribution. This noise is also visible in the profile.


## Using a time-variant window

The use of time-variant filtering for attenuation compensation introduces the possibility of selecting a time-variant window to regularise the compensation based on the local frequency content of the signal. This is useful, because high-frequencies generated by deeper features will undergo more attenuation before reaching the detector compared to shallow features, as the waves have travelled further. This means that high-frequency components from shallow features (which appear early in the time signal) may be above the noise floor, while the same frequency components from deeper features (which appear later in the time signal) may not. The use of a time-variant window to regularise the attenuation compensation means the filter can adapt to the local frequency content of the recorded signal.

If the optional input `'FilterCutoff'` is not specified, the function `attenComp` automatically chooses a time-variant cutoff frequency for the window based on the average time-frequency distribution of the signals. The steps for computing the cutoff frequency are detailed in the documentation for `attenComp`. An image of the average time-frequency distribution of the sensor data along with the extracted time-variant cutoff frequency before (yellow) and after (white dashed) smoothing are shown below. It is clear from this example (shown with a dynamic range of 40 dB) that the frequency content of the signals changes over time.

The reconstructed photoacoustic image after attenuation compensation using a window with a time-variant cutoff frequency is shown below. Again, compared to the uncompensated case, the reconstructed image is much sharper, and the magnitude of small features is closer to their expected values. Compared to the image after compensation using a fixed cutoff frequency of 3 MHz, the reconstructed image shown below is not as sharp. However, the noise has now been significantly suppressed.


## Correcting the signals individually

Correcting the signals using the average time-frequency distribution has a distinct computational advantage in that the time-varying cutoff frequency and the time-variant filter only need to be calculated once. This makes it reasonably fast to apply, even to a large number of signals. However, the downside is that the time-varying cutoff frequency may not be optimal for each individual signal. In the reconstructed image shown above, this results in some artefacts in the reconstructed image, most noticeably at the inside bottom edge of the phantom.

As an alternative, it is also possible to apply attenuation compensation to the recorded time signals individually by calling `attenComp` in a loop. This way, the cutoff frequency is calculated based on the time-frequency distribution of the individual time series. As the loop operations are completely independent, it is possible to parallelise the calculations using the parallel computing toolbox (set the flag `USE_PARALLEL_COMPUTING_TOOLBOX` to `true` in the example .m file).

```
% correct for acoustic attenuation using time variant filtering regularised
% by a Tukey window with a time-variant cutoff frequency based on the
% time-frequency distribution for each signal
sensor_data_comp = zeros(size(sensor_data_lossy));

if ~USE_PARALLEL_COMPUTING_TOOLBOX
    
    % loop through signals
    for index = 1:size(sensor_data_lossy, 1)

        % correct signal
        sensor_data_comp(index, :) = attenComp(sensor_data_lossy(index, :), kgrid.dt,...
            medium.sound_speed, medium.alpha_coeff, medium.alpha_power, 'FrequencyMultiplier', 3);

    end

else
    
    % start matlab pool
    parpool;
    
    % loop through signals using parfor
    parfor index = 1:size(sensor_data_lossy, 1)
        
        % correct signal
        sensor_data_comp(index, :) = attenComp(sensor_data_lossy(index, :), kgrid.dt,...
            medium.sound_speed, medium.alpha_coeff, medium.alpha_power, ...
            'FrequencyMultiplier', 3, 'DisplayUpdates', false); 
        
    end
    
    % delete matlab pool
    delete(gcp('nocreate'));
    
end
```

The reconstructed image after attenuation compensation is shown below. The reconstructed image is much sharper without noticeable noise or artefacts, and the magnitude of the edges has been restored. The primary drawback of correcting the signals individually is that calculating the time-varying cutoff frequency and constructing the time-variant filter for each individual signal is computationally more expensive than using an average or fixed cutoff frequency.
